# Are females in classical hematology getting a fair share? Uncovering gender disparities in NIH R01 grants

**DOI:** 10.3389/fsoc.2024.1430369

**Published:** 2024-11-18

**Authors:** Sara Khan, Faraz Eshaghi, Mohammed Z. Rehman, Serena Kotwal, Mariya Syed, Kainat Khan, Kapisthalam S. Kumar

**Affiliations:** ^1^HCA Healthcare/USF Morsani College of Medicine GME, Hudson, FL, United States; ^2^Edward Via College of Osteopathic Medicine, Auburn, AL, United States; ^3^Florida Cancer Specialists, Trinity, FL, United States

**Keywords:** classical hematology, research funding, gender representation, NIH RePORTER, NIH R01 grants

## Abstract

Gender Disparity remains a pressing issue in academic medicine, notably in classical hematology where females continue to be underrepresented by the National Institutes of Health (NIH) for funded R01 grants. In this research, we analyzed ten years of NIH R01 grants funded in classical hematology, covering the period from 2012 to 2022. Of the 250,031 R01 grants funded during this period, females received only 32.9%. Further breakdown of the data by different NIH institutes highlights varying degrees of gender gaps, with specific institutes showing pronounced disparities. While some NIH Institutes have made progress in bridging the gap, others lag, indicating a need for a closer examination of institutional practices. We found that despite modest advancements, less than 50% of R01 grants were funded to females. These findings underscore persistent gender inequity and require concerted efforts to create a more inclusive atmosphere supportive of women’s progress in academic medicine.

## Introduction

There has been a historical underrepresentation of women in the field of clinical (Patel et al., 2021; Jacobs et al., 2022) and academic (Carr et al., 1993) hematology. Women in medicine also have lower salaries (Carr et al., 1992; Jagsi et al., 2012), fewer publications (Jagsi et al., 2006; Duma, 2020; Holliday et al., 2014), and less research funding (Jagsi et al., 2009; Ley and Hamilton, 2008). Publication is an important metric in academic medicine, and the number of publications trend in favor of male authors. Several studies suggest that female faculty members are less likely to publish papers compared to their male colleagues (Jagsi et al., 2006; Tesch et al., 1995; Barnett et al., 1998). More than one-half of cardio-vascular trials published in 3 high-impact journals between 2014 and 2018 lacked women investigators on their executive committees (Denby et al., 2020; van Spall et al., 2021). Thus, gender inequality is a major concern in academic medicine.

The National Institutes of Health (NIH) is a major funding source for biomedical research, playing a pivotal role in shaping the scientific landscape. However, a disconnected pattern persists in the distribution of NIH grants in the hematology research domain. Herein, we present a ten-year retrospective analysis of the Research Project Grants (R01) awarded by the National Institutes of Health (NIH) across the fiscal years 2012–2022. Our temporal analysis examined gender disparities in the field of non-malignant hematology research. Our examination analyzed existing data on NIH grant distribution, shedding light on the underrepresentation of women on funded projects. In this paper we aim to provide data to support and extend a broader conversation on gender inequality within the scientific community and advocate for changes that promote a more inclusive and supportive environment for all researchers, irrespective of gender, to excel in the pursuit of advancing hematology research.

## Methods

In the conduct of this study, we adhered to the Strengthening the Reporting of Observational Studies in Epidemiology (STROBE) reporting guidelines. Institutional review board approval was not required since the data were publicly available and did not use patient records. Data were extracted for the fiscal years 2012–2022, and we focused exclusively on active R01 grants awarded and categorized by several NIH Institutes. We compiled a list of classical hematology related MESH terms that allowed us to search for classical hematology R01 grants. MESH terms related to classical hematology were identified using the National Library of Medicine (NLM) database. Terms that pertained to classical hematology such as anemia, coagulation disorders, and hemophilia were included. Terms that were exclusively associated with malignant hematology were excluded. The data were leveraged using the NIH RePORTER tool, Tidyverse, and janitor packages in R. The R script used the “repoRter.nih” library with the NIH API, specifically the NIH RePORTER tool. The NIH RePORTER tool facilitates the extraction of data from the NIH database. The dataset incorporated key grant parameters such as grant ID, agency code, activity code, abstract text, project title, fiscal year, activity status, award amount, organization, and principal investigator’s (PI) name.

In order to distinguish a PI’s gender, the PI’s first names were processed using the gender package in R, which provided a gender-wise distribution of recipients. The gender package in R was employed to determine the likely gender of Principal Investigators (PIs) based on their first name. R package uses historical datasets from the US Social Security Administration and the U.S. Census Bureau via Integrated Public Use Microdata Series (IPUMS). The first names of authors are extracted from the dataset and the package then matches these names to the mentioned databases and assigns a gender based on the probability derived from the frequency of the name being associated with a particular gender in the data. For statistical analyses, the proportions of females with accepted active R01 grants were compared between 2012 to 2022; this proportion analysis was also done among each NIH Institute. Further, linear regression and associated statistical tests were used to identify whether there was a significant change in grants received by either gender over this time period.

## Results

**Figure 1:**
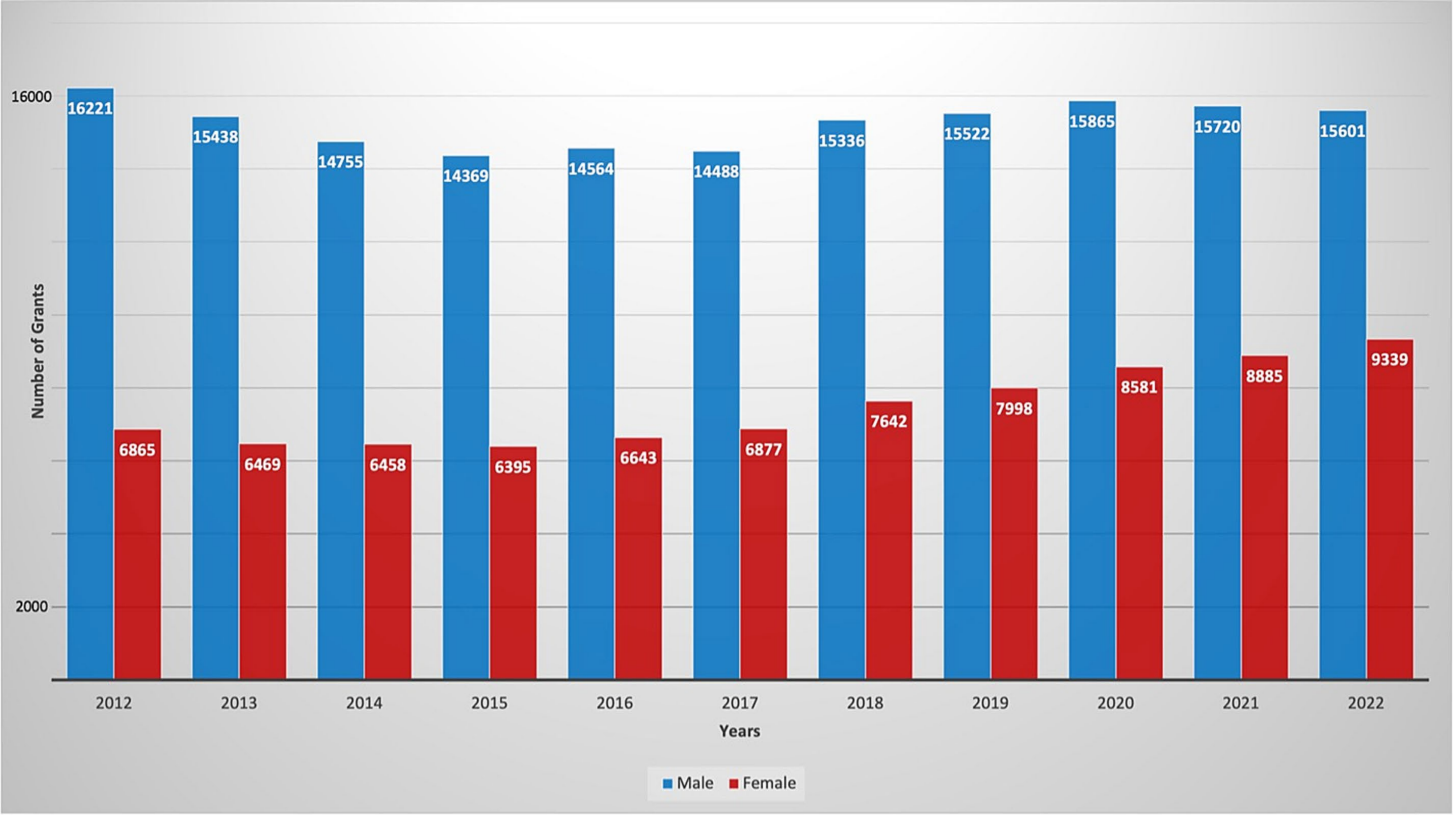
Number of active NIH R01 hematology grants distributed by gender, throughout the years 2012–2022.

**Figure 2:**
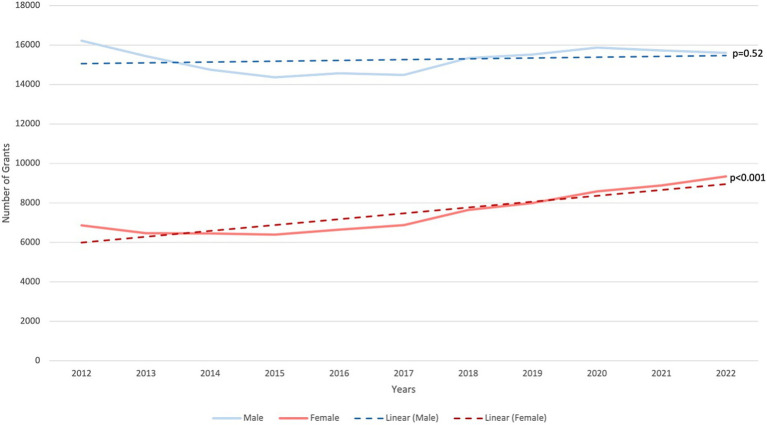
Trend (linear) showing gender disparity in NIH R01 hematology grants, throughout the years 2012–2022.

**Figure 3:**
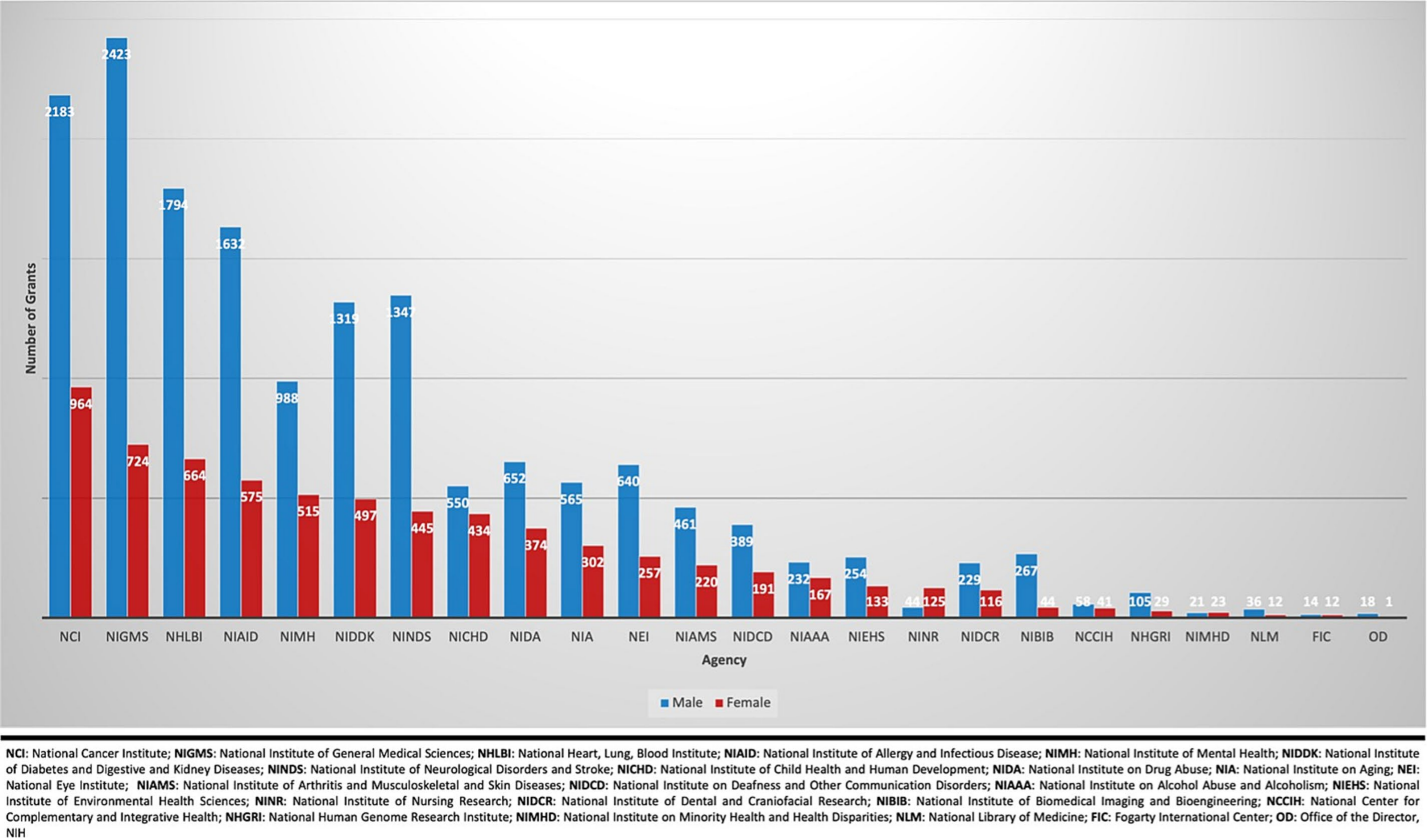
Allocation of active NIH R01 classical hematology grants by NIH institution in 2012.

**Figure 4:**
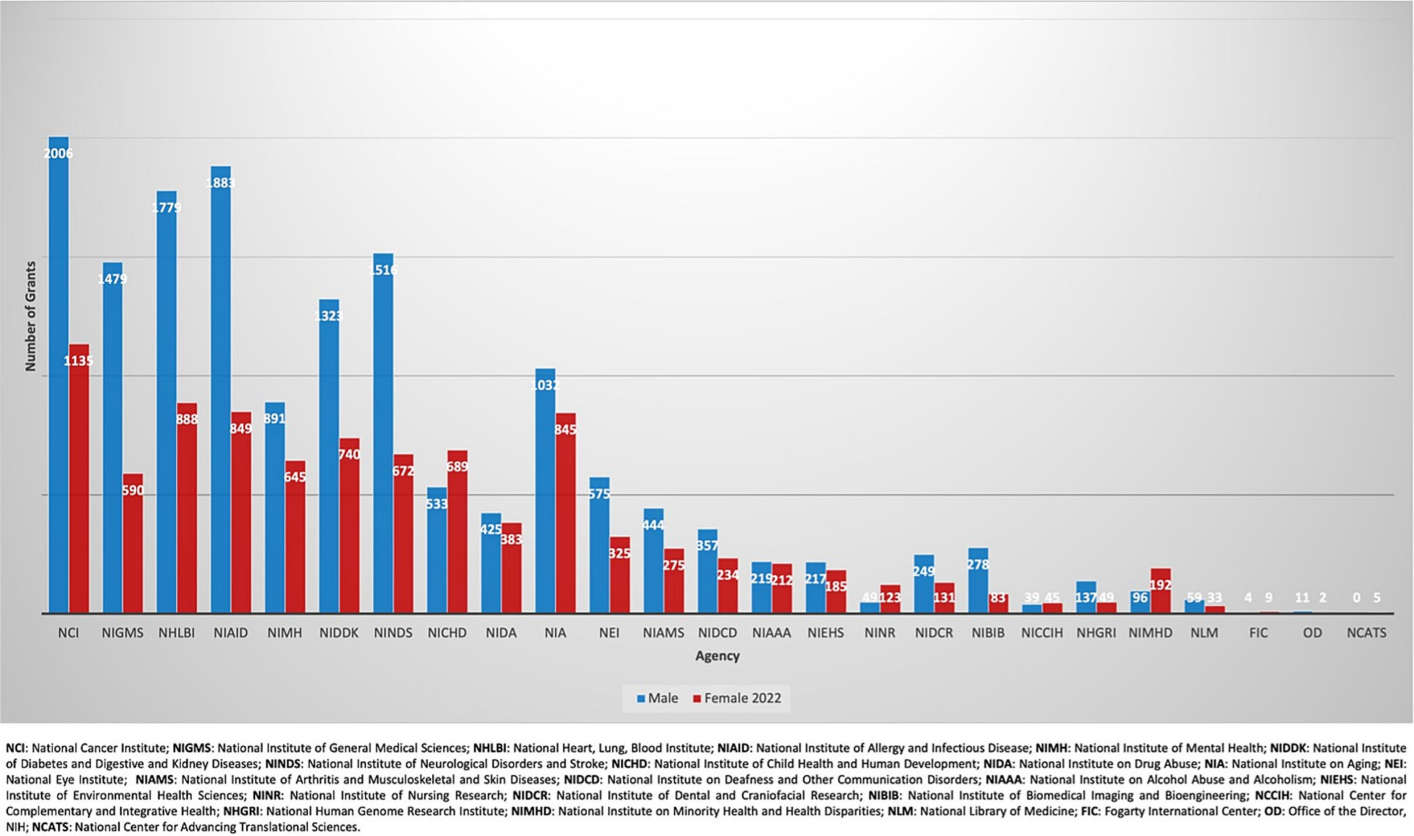
Allocation of active NIH R01 classical hematology grants by NIH institution in 2022.

A total of 250,031 active R01 grants were funded by the NIH during the fiscal years spanning 2012 to 2022 (Figure 1). Females (*n* = 82,152: 32.9%) received fewer R01 grants than males (*n* = 167,879: 67.1%). From 2012 to 2022, there was no significant change in R01 grants funded to males as there were 16,221 grants awarded in 2012 (70.3%, CI: [0.65–0.76]) and 15,601 grants in 2022 (62.6%, CI:[0.56–0.69]) with a *p*-value = 0.52 (Figure 2). In contrast, there was a significant increase in the number of females funded on R01 grants from 6,865 grants (29.7%, CI:[0.29–0.30]) to 9,339 grants (37.7%, CI:[0.37–0.38]) with a *p*-value <0.001, indicating a noteworthy increase (Figure 2). Further categorization and analysis were conducted based on the various NIH Institutes and Centers (Figures 3, 4). In 2012, the National Institute of General Medical Sciences (NIGMS), National Institute of Neurological Disorders and Stroke (NINDS), and National Institute of Biomedical Imaging and Bioengineering (NIBIB) awarded fewer than 25% of the total grants to females (Figure 3). By 2022, the NIBIB had the most extensive gender gap with females being awarded only 23% of grants with a *p*-value <0.001 (Figure 4). In contrast, the National Institute of Minority Health and Disparities (NIMHD) and the National Institute of Nursing Research (NINR) were the only agencies in 2012 to award more grants to females with approximately 52% (*p* < 0.001) and 74% (*p* < 0.001) of grants, respectively (Figure 3). In 2022, the NINR and NIHMD still had more grants awarded to females versus males (Figure 4). In addition to those agencies, by 2022, the National Institute of Child Health and Human Development (NICHD) and National Center for Complementary and Integrative Health (NCCIH) joined the NINR and NIHMD awarded more than 50% of grants to females (Figure 4). The National Heart, Lung, and Blood Institute (NHLBI) which funds a large amount of non-malignant hematology grants, was noted to fund 2,458 R01 grants in 2012 (Figure 3). Of these 2,458 R01 grants, only 664 (27%, CI:[0.25–0.29]) R01 grants were awarded to females. Whereas in 2022, despite an increase in the total number of R01 grants funded by the NHLBI to 2,667, only 888 (33.3%, CI:[0.32–0.35]) R01 grants were awarded to females, a marginal improvement (*p* < 0.001) (Figure 4).

## Discussion

Our analysis of classical hematology research funding revealed that, from the fiscal years 2012 to 2022, 33% of total active R01 grant awardees were female. Surprisingly, we did not observe a significant increase in grants funded to females when comparing 2012 to 2022. In 2012, females were funded 29.7% of total classical hematology grants from the NIH. By 2022, the percentage of grants funded to females under classical hematology increased to 37.4%. On the other hand, the number of active R01 grants awarded to males remained stable from 2012 to 2022. The positive trajectory observed for females may potentially be due to heightened awareness, advocacy efforts, or changes in NIH policies fostering gender equity. Organizations such as the Women in Hematology Work Group of the American Society of Hematology and Research and Practice in Thrombosis and Haemostasis are working hard to promote gender equity and provide career development opportunities, community support, and scholarships for women in hematology (Liblik et al., 2022). However, despite the marginal increase in R01 grants funded to females over the past decade, females still secure fewer than 50% of R01 grants. Further investigation of contributing factors is imperative to create targeted efforts to bridge this persistent gap.

When breaking down the data by specific NIH Institutes/Centers, notable disparities emerged. The National Institute of Biomedical Imaging and Bioengineering (NIBIB) funded the lowest number of female awardees for NIH R01 grants in classical hematology. Both in 2012 and in 2022, NIBIB awarded less than 25% of classical hematology R01 grants to females (Figures 3, 4). These findings necessitate closer examination to understand the unique challenges faced by female researchers within this institution. Conversely, NIH institutions such as the National Institute of Minority Health and Disparities (NIMHD) and the National Institute of Nursing Research (NINR) consistently award a higher proportion of NIH R01 grants to females. This positive trend suggests that certain NIH institutions have been successful in promoting gender diversity. Identifying practices and policies implemented by the NINR and NIMHD could potentially provide valuable insights for developing strategies to address gender disparities that are being noted in other NIH institutions. Turning attention to the National Heart, Lung, and Blood Institute, which funds a significant number of classical hematology grants, our analysis reveals a concerning gender disparity in the distribution of R01 grants in this field. Despite a marginal increase in R01 grants funded to females from 2012 to 2022 at this Institute, an increase from 27% in 2012 to 33.3% in 2022 signifies a relatively modest improvement. The fact that less than 50% of R01 grants are awarded to females in this institution within this timeframe underscores a persistent gender gap. Although we acknowledge the positive trajectory from 2012 to 2022, this disparity emphasizes the urgent need for targeted interventions to address root causes that hinder a more equal distribution of grants to female principal investigators.

The observed pattern of fewer female awardees by NIH in classical hematology is mirrored across various specialties including gastroenterology, cardiology, oncology, urology, general surgery, dermatology, radiology, and internal medicine making this a persistent and systemic issue (Khan et al., 2024; Eshaghi et al., 2023; Shahid et al., 2022; Cheng et al., 2016; Berg and Ashurst, 2019a; Hakam et al., 2021; Jutras et al., 2022; Berg et al., 2021). Particularly noteworthy is the field of cardiology, where a study revealed that females secured 20% of NIH R01 grants from the fiscal years 2011 to 2020 (Shahid et al., 2022). Similar to our findings, there was a positive trajectory marked by an increase in R01 grants recently awarded to females in cardiology (Shahid et al., 2022). However, a simultaneous rise in grants awarded to males, contrary to the observed classical hematology pattern, indicates that while progress is evident, there remains work to be done to expedite the closing of this gender gap. Despite several specialties documenting gender disparities in NIH R01 funding, family medicine serves as an exception. Family medicine is the first reported specialty to achieve gender parity in the distribution of NIH R01 grant funding (Berg and Ashurst, 2019b). These encouraging advancements highlight the pivotal role of developing initiatives driving advancements in gender equity in the allocation of research funding.

Throughout much of the 20th century, medical schools and residency programs were dominated by male students and faculty; a historic trend that persists in senior faculty positions in hematology and oncology to this day. There can be a considerable time lag before changes in gender roles are fully realized in workforce demographics. The academic year 2004–2005, was the first time female medical school applicants were more than male applicants (50.8% vs. 49.2%) (AAMC, n.d.). However, this only lasted for 2 years, and male applicants remained the majority, until the academic year 2018–2019 (AAMC, n.d.). In discussing the gender disparity in medicine, an important limitation to consider is whether the lack of representation is entirely due to external barriers or rather a result of personal choices made by females. Societal and cultural norms spanning generations have led women across all fields to continue to select jobs that accommodate work-family balance at the expense of forgone wages and delayed career advancement (Jovic et al., 2006). It is also important to keep in mind the context and the time required for societal changes to translate into tangible shifts in workforce dynamics.

The demanding nature, long hours, and high-pressure environments of academic medicine can disproportionately affect women, particularly those trying to balance family responsibilities (Gaudet et al., 2022). These challenges could lead to women choosing not to pursue academic positions, or leave their current positions (Martinez et al., 2007). Several studies have shown that family demands and self-confidence, are factors that hinder women from pursuing a position of Independent or Principal Investigator (Martinez et al., 2007; Kaplan and Granrose, 1993; Sears, 2003; Mason and Goulden, 2004; Hoonakker et al., 2005). Academic and research institutions may perpetuate cultural norms and biases that impact women’s careers. Implicit biases in hiring and promotion can disadvantage women. Search committees may unconsciously favor male candidates (Wynn and Correll, 2018) or devalue the accomplishments of women due to gender stereotypes (Ellemers, 2018). Male-dominated networks can exclude women, limiting their opportunities for collaboration and professional development. One of the most important barriers to women leading clinical trials is the systemic sexism that under-recognizes their academic accomplishments. It has been reported that women receive less research funding than men, and research submitted under a woman’s name is likely to receive a more critical review than the same research under a man’s name (Budden et al., 2008). Studies have shown that gender stereotypes can influence decision-making processes, which could prove to be a disadvantage for women applicants (Carnes et al., 2005; Sato et al., 2021).

Institutions should incorporate bias training programs to raise awareness and promote fair evaluation processes in grant applications and research proposals. Provide comprehensive training for grant reviewers to recognize and mitigate implicit biases that may influence their evaluations and also implement anonymous review processes where possible to minimize gender bias by focusing solely on the quality of the research proposal rather than the identity of the applicant. We also suggest that regular audits be conducted for funding allocation to ensure that grants are being distributed equitably among male and female researchers. We also suggest the deidentification of grant applicants’ data, which could eliminate the bias entirely. This was done in an experimental study in 2019, that showed little to no race or gender bias in the initial R01 evaluations, and any remaining bias was negligible in size (Forscher et al., 2019).

The absence of strong mentorship and support systems, coupled with limited networking opportunities also limits the progression of women in this field. The scarcity of women in academic and editorial roles in hematology and other fields of medicine (Amrein et al., 2011) may contribute to the lack of role models and mentors. Mentorship is crucial for overcoming the disparity and can provide guidance, support, and networking opportunities crucial for career advancement. Successful mentorship programs like, “Mentornet” (Chin et al., 2003), “ELAM program” (Jagsi and Spector, 2020), “Georgia Tech Advance Program” (Fox, 2003), “UC Davis BIRCWH Program” (Bauman et al., 2014), and “UNC Working on Women in Science” (Shoemaker et al., 2016) are some great examples and, we believe institutions should actively work towards establishing mentorship programs that specifically address the needs of women researchers. Providing leadership training and opportunities for women can help prepare them for senior roles and increase their representation in decision-making positions. Promoting a culture of inclusion and respect within academic medicine is essential. This can be achieved through diversity and inclusion initiatives mentioned above, zero-tolerance policies for harassment and discrimination, and efforts to highlight and celebrate the achievements of women researchers in hematology through awards, public recognition, and media campaigns to inspire and encourage future generations.

We acknowledge that our study has limitations. Given that this is a retrospective study, the search terms used during the data-acquiring process are crucial to shaping the study’s inclusion and exclusion criteria. Using terms that are not comprehensive raises concern for excluding relevant studies. Additionally, using terms that are biased towards certain types of studies can lead to missing important unpublished negative results. Such outcomes can perpetuate publication bias. Another limitation of our study is the inability to account for all grant applications, as this information is not publicly available on NIH RePORTER. Consequently, we can only provide data on grant awards and not on the application themselves. Furthermore, using first names to infer gender can introduce biases due to cultural variations, unisex names, etc. Additionally, relying on publicly available data can result in selection bias, as it may not be comprehensive and often omits unpublished negative findings. Lastly, this study focuses on quantitative data which limits its ability to explore the underlying causes of gender disparities in NIH grant funding within hematology. Without qualitative insights, the study cannot fully capture the sociocultural factors that may contribute to these disparities.

In summary, our analysis revealed a significant disparity in the proportion of male and female NIH R01 grant awardees. These disparities improved marginally at some NIH Institutes over the decade long data comparison, however, disparities persisted for several institutes, including those responsible for a significant portion of funding in classical hematology. Addressing the underrepresentation of women in hematology is a multifaceted challenge that can be overcome through collaborative efforts from institutions, policymakers, and the scientific community. Spreading awareness about the importance of work-life balance is key to improving the well-being, productivity, and overall quality of life for women in the workplace. Establishing a network where women in leadership roles across all fields can represent themselves and engage in conversation about their own experiences and challenges would promote solidarity and perhaps even encourage women to pursue more opportunities for career growth. By understanding the causes, acknowledging barriers, and implementing targeted solutions, we can create an inclusive environment that allows women to thrive in the field of hematology, ultimately contributing to advancements in the field of medicine and improving healthcare outcomes for all.

## Data Availability

Publicly available datasets were analyzed in this study. This data can be found here: https://reporter.nih.gov/.
